# The relationship between major dietary patterns and fertility status in iranian men: a case–control study

**DOI:** 10.1038/s41598-021-98355-4

**Published:** 2021-09-22

**Authors:** Farahnaz Haeri, Makan Pourmasoumi, Reza Ghiasvand, Awat Feizi, Amin Salehi-Abargouei, Laleh Dehghan Marvast, Cain C. T. Clark, Masoud Mirzaei

**Affiliations:** 1 Department of Community Nutrition, School of Nutrition and Food Science, Isfahan University of Medical Sciences, Isfahan, Iran; 2 Gastrointestinal and Liver Diseases Research Center, Guilan University of Medical Sciences, Rasht, Iran; 3 Food Security Research Center, Isfahan University of Medical Sciences, Isfahan, Iran; 4 Department of Epidemiology and Biostatistics, School of Public Health, Isfahan University of Medical Sciences, Isfahan, Iran; 5 Nutrition and Food Security Research Center, Department of Nutrition, School of Public Health, Shahid Sadoughi University of Medical Sciences, Yazd, Iran; 6 Andrology Research Center, Yazd Reproductive Sciences Institute, Shahid Sadoughi University of Medical Sciences, Yazd, Iran; 7 Centre for Intelligent Healthcare, Coventry University, Coventry, CV1 5FB UK; 8 Yazd Cardiovascular Research Centre, Shahid Sadoughi University of Medical Sciences, Yazd, Iran

**Keywords:** Health care, Health occupations

## Abstract

In this case–control study, we aimed to investigate the association between major dietary patterns and fertility status in Iranian men. The study population included 400 newly diagnosed infertile men and 537 healthy individuals without a history of infertility in Yazd, Iran. Infertility was confirmed clinically, based on the World Health Organization (WHO) criteria. Dietary intake was assessed using a 168-item semi-quantitative food frequency questionnaire (FFQ), and dietary patterns were determined based on a principal component analysis. Four major dietary patterns were found in this study, including healthy, Western, mixed, and traditional dietary patterns. After adjustments for potential confounders, men above the median of a healthy dietary pattern showed a reduced risk of infertility compared to those below the median (OR 0.52; 95% CI 0.33–0.83). In contrast, men with greater adherence to Western and mixed dietary patterns were more likely to be infertile (OR 2.66; 95% CI 1.70–4.17 and OR 2.82; 95% CI 1.75–4.56, respectively). Also, there was no significant association between the traditional dietary pattern and the odds of infertility. The present study suggests that greater adherence to a healthy dietary pattern may have an inverse association with the odds of infertility; however, Western and mixed dietary patterns may be associated with an increased risk of infertility.

## Introduction

Infertility is a common global problem with a prevalence of approximately 15%. It is broadly defined as a failure to conceive despite frequent unprotected intercourse for at least one year^1^. Infertility is one of the most important stressors affecting couples^1^, with more than 50% of infertility cases attributed to male fertility disorders^2^. One of the main causes of male infertility is a decrease in semen quality^3^. Abnormalities in semen parameters typically include azoospermia, oligospermia, and abnormalities in semen morphology, motility, and volume and sperm concentration^4^. There are several putative reasons for a decline in semen quality; however, environmental pollution, stress and anxiety, and unhealthy eating habits are the main contributors^5,6^.

The literature suggests that there is an association between the intake of various nutrients and male sexual health^7^. It seems that food intake and generally diets are related to fertility and semen parameters, both directly and indirectly. The favorable effects of foods containing antioxidants on the improvement of conventional semen parameters and sperm function have been previously documented^8,9^. Moreover, some studies have demonstrated a relationship between obesity^10^ and depression^11^ and the risk of infertility among men. Considering the vital role of diet in both obesity and depression, it may also have a potential indirect effect on male infertility.

The potential association between nutrition and male infertility has attracted increasing attention in recent years. While some researchers have explored the possible association between a single food or food group and fertility parameters^12–16^, others have investigated the relationship between dietary patterns as a whole and infertility and/or abnormalities in semen quality markers^17–19^. Despite the importance of information provided by a single nutrient/food analysis, evaluation of the dietary pattern can yield an integrated estimate of both contribution and synergistic effects of some factors and may be used to describe different aspects of a diet^20,21^.

Besides, dietary patterns are inherently related to specific cultural, religious, ethnic, and regional backgrounds. Therefore, studies conducted in other countries cannot adequately describe the nutritional status of the Iranian population. So far, few studies have been performed on only asthenozoospermia, and its association with food groups, fatty acids, and nutrient patterns has been investigated in Iranian populations^22–24^; therefore, studies with a large sample size, assessing the possible association between diet and male infertility, are still warranted. The present study aimed to investigate the association between major dietary patterns and male infertility status in a large Iranian population.

## Results

### General characteristics and dietary intake of the participants

The demographic, anthropometric, and total energy intake data of the participants are presented in Table 1. There was no significant difference in terms of BMI, weight, or education years between the case and control groups. However, waist and hip circumferences, as well as socioeconomic scores, were significantly higher in the control group as compared to the case group (P < 0.001). Similarly, the percentages of people with the lowest level of physical activity and current smokers were significantly higher in the control group as compared to the case group (P < 0.001). In contrast, the percentage of pesticide use and the amount of energy intake were significantly higher in the case group as compared to the control group (P < 0.001). Besides, there was a significant difference in the consumption of almost all predefined food groups between the case and control groups, except for poultry, eggs, whole grains, and animal fat (Supplementary Table 1).

**Table 1:** The characteristics of Iranian men in the case and control groups (number [%] unless otherwise stated).

Characteristics	Case (n = 400)	Control (n = 537)	P-value*
Age (years)	33.66 ± 6.35	34.49 ± 6.52	0.25
BMI (kg/m^2^)	26.1 ± 5.33	27.3 ± 4.78	0.46
Weight (kg)	79.4 ± 18.36	81.7 ± 15.67	0.23
Waist circumference (cm)	93.6 ± 20.01	94.3 ± 12.79	< 0.001
Hip circumference (cm)	96.8 ± 21.80	101.3 ± 7.10	< 0.001
Waist-to-hip ratio	1.08 ± 0.17	1.07 ± 0.30	0.52
Socioeconomic status (SES)**	4.5 ± 1.97	5.6 ± 1.37	< 0.001
Education (years)	10.5 ± 4.30	10.7 ± 3.90	0.17
**Smoking history**			
Current smoker (%)	138 (34.5%)	192 (33.3%)	< 0.001
Non-smoker (%)	162 (40.5%)	313 (54.2%)	
Ex-smoker (%)	100 (25%)	72 (12.5%)	
**Use of pesticides**			
Yes (%)	129 (32.2%)	56 (9.3%)	< 0.001
No (%)	271 (67.8%)	546 (90.7%)	
Total energy intake (kcal/day)	3001 ± 659	2547 ± 530	< 0.001
**Physical activity level**			
Inactive (%)	136 (34%)	169 (28%)	< 0.001
Moderate activity (%)	157 (39.2%)	166 (27.5%)	
Active (%)	107 (26.8%)	269 (44.5%)	

Data are presented as mean ± SD or percentage for continuous or categorical data, respectively.

*P-value was determined using independent t-test for quantitative variables and Chi-square test for categorical variables.

**SES was analyzed according to the following variables: residential status (landlord/tenant), use of a washing machine or dishwasher (yes/no), number of international travels, having a car (yes/no), occupation, and education (years of study).

### Major dietary patterns

As shown in Table 2, four dietary patterns, explaining 44.43% of variance, were extracted and labeled as Western diet, healthy diet, traditional diet, and mixed diet. In the Western diet which explained 11.37% of variance, the intake of red meat, processed meat, fast food, legumes, and refined grains was high. The healthy diet, which explained 18.05% of variance, was defined as a high intake of fish, eggs, dairy products, fruits, vegetables, nuts, salty snacks, vegetable oils, condiments, and pickles. The traditional diet, which explained 8.47% of variance, was enriched with carbonated drinks, potatoes, whole grains, sugars, sweets, deserts, tea, and coffee. Finally, the mixed diet, explaining 6.52% of variance, referred to the high consumption of organ meat and animal fat.

**Table 2:** The factor loading matrix for five major dietary patterns identified by the principal component approach (PCA).

Food groups	Dietary pattern			
	Western diet	Healthy diet	Traditional diet	Mixed diet
Red meat	0.624	–	0.275	–
Processed meat	0.830	–	− 0.209	–
Organ meat	–	–	–	0.775
Fish and other seafood	0.343	0.724	–	–
Poultry	–	–	–	–
Fast food	0.740	–	–	0.468
Eggs	− 0.348	0.548	–	–
Carbonated drinks	–	0.252	0.306	–
Dairy products	–	0.345	–	–
Fruits and dried fruits	–	0.619	–	–
Vegetables	–	0.658	0.213	0.241
Potatoes	–	–	0.508	–
Legumes	0.802	–	–	–
Nuts	–	0.415	–	–
Whole grains	–	− 0.226	0.427	–
Refined grains	0.465	0.306	–	− 0.363
Salty snacks and vegetables	0.223	0.728	–	–
Animal fat	–	–	–	0.543
Vegetable oils	–	0.533	–	0.381
Olives	–	–	–	–
Sugar, sweet, and desserts	–	–	0.774	–
Condiments and pickles	0.299	0.695	–	–
Tea and coffee	–	–	0.688	–

Factor loadings <|0.20| were excluded.

### Relationships between dietary patterns and male infertility

The multivariate adjusted odds ratios (OR) and 95% confidence intervals (CIs) across the median of dietary patterns are presented in Table 3. Each dietary pattern score lower than the median was considered as the reference level. The results showed that men with greater adherence to a healthy dietary pattern had a reduced risk of infertility (OR 0.64; 95% CI 0.50–0.83). After multiple confounders were considered, this association remained significant (OR 0.52; 95% CI 0.33–0.83). In contrast, there was a non-significant direct association between greater adherence to a Western dietary pattern and the risk of infertility in the unadjusted model (OR 1.11; 95% CI 0.86–1.43). However, after controlling for potential confounders, this association became significant, as the participants above the median with the Western dietary pattern had a higher risk of infertility compared to those below the median (OR 2.66; 95% CI 1.70–4.17).

**Table 3:** Multivariable adjusted odds ratio (OR) and 95% confidence interval (CI) for infertility in tertiles of dietary pattern scores.

Dietary pattern	Adjusted models			
	Crude model	Model I	Model II	Model III
**Healthy diet (18.05% of variance)**				
> Median (Case: 174; Control: 328)	0.64 (0.50–0.83)	0.90 (0.64–1.28)	0.92 (0.64–1.34)	0.52 (0.33- 0.83)
< Median (Case: 226; Control: 276)	1	1	1	1
**Western diet (11.37% of variance)**				
> Median (Case: 206; Control: 295)	1.11 (0.86–1.43)	1.09 (0.78–1.52)	1.34 (0.93–1.92)	2.66 (1.70–4.17)
< median (Case: 194; Control: 309)	1	1	1	1
**Mixed diet (6.52% of variance)**				
> Median (Case: 303; Control: 191)	6.75 (5.07–8.98)	7.19 (4.94–10.45)	7.36 (4.87–11.04)	2.82 (1.75–4.56)
< Median (Case:97; Control: 413)	1	1	1	1
**Traditional diet (8.47% of variance)**				
> Median (Case: 223; Control: 277)	1.48 (1.15–1.91)	1.89 (1.34–2.66)	1.50 (1.03–2.18)	1.36 (0.86–2.16)
< Median (Case: 177; Control: 327)	1	1	1	1

Model I: Adjusted for age, BMI, and physical activity.

Model II: Adjusted for model I plus education, use of pesticides, smoking, and socioeconomic status (SES).

Model III: Adjusted for model II plus energy.

Interestingly, men with greater adherence to a mixed dietary pattern had a higher risk of infertility compared to those with lower adherence (OR 6.75; 95% CI 5.07–8.98); this association remained significant after adjustment for potential confounders (OR 2.82; 95% CI 1.75–4.56). Although men above the median of the traditional dietary pattern had a 46% increased risk of infertility (OR 1.48; 95% CI 1.15–1.91), this association became insignificant after controlling for confounders (OR 1.36; 95% CI 0.86–2.16).

## Discussion

In this cross-sectional study, we found that greater adherence to a healthy dietary pattern was associated with the reduced risk of infertility. Moreover, a significant direct association was found between Western and mixed dietary patterns and the risk of infertility. Although our findings were similar to the traditional dietary pattern, which was associated with an increased risk of infertility, the relationship was insignificant in the fully adjusted model.

In recent years, our understanding of the etiology of male infertility has increased substantially; however, major improvements are still needed to increase awareness and improve treatments^25,26^. Lifestyle factors, such as diet, have been implicated in male infertility. They may play a significant role in the onset/progression of disease, concomitant with genetic and idiopathic factors^27^. The present study highlighted that greater adherence to a healthy diet yields a significant decrease (48%) in the risk of infertility. In line with our study, Jurewicz et al.^19^ indicated that adherence to a Prudent dietary pattern (similar to the healthy dietary pattern in our study) was associated with a higher sperm concentration and improved sperm motility. Oostingh et al.^28^ also demonstrated that adherence to a healthy diet, consisting of large amounts of olive oil, seafood, and vegetables, had a significant positive effect on the quality of semen parameters, which is consistent with our results.

In contrast, in a cross-sectional study conducted on 114 men aged 20–55 years from Poland, no significant association was found between adherence to a healthy dietary pattern and several sperm parameters, including progressive motility, total motility, total sperm count, sperm concentration, and morphology^29^. This discrepancy may be attributed to differences among foods/food groups between the two studies; for instance, fish and seafood, which cause improvements in sperm quality due to their polyunsaturated fatty acid (PUFA) contents, were not included in the healthy dietary pattern of the mentioned study, unlike our study. The healthy diet in our study was rich in fish, dairy products, eggs, vegetables, fruits, nuts, and vegetable oils. According to available evidence, a higher seafood intake can result in elevated levels of eicosapentaenoic acid (EPA) and docosahexaenoic acid (DHA) in sperm cells and seminal plasma. Also, PUFAs in the spermatozoa membrane can affect the sperm motility. A strong association has been shown between the sperm membrane DHA levels and sperm motility^30,31^.

Another study reported that a dietary vegetable intake is important for the improvement of semen quality. Generally, vegetables are rich in phenols, antioxidants, and magnesium^32,33^, and strong evidence suggests that antioxidants are positively associated with sperm and seminal parameters^34,35^; high contents of fiber sources, such as vegetables and fruits, can also contribute to reduced estrogen levels^23,36^. Besides, a healthy dietary pattern is rich in B12, folate, and antioxidants; folate is a particularly critical nutrient for methylation, stability, and DNA replication.

DNA methylation is a biochemical process involved in normal cellular development and differentiation^37^. It has been demonstrated that anomalies in sperm DNA methylation and homocysteine accumulation occur in men with infertility. Regarding the high levels of folate and B12 in people with a healthy dietary pattern and the abovementioned mechanisms, the healthy dietary pattern may help prevent DNA damage and improve the sperm concentration and motility^36,38^. Besides, oxidative stress levels can affect sperm motility and increase DNA fragmentation; therefore, the intake of antioxidants can increase the sperm concentration and improve its progressive motility^37,39^.

Another important finding of our study is that adherence to a Western dietary pattern increased the risk of infertility. Accumulative evidence suggests that adherence to a Western diet is associated with an increased risk of infertility. In line with our findings, a cross-sectional study on 7282 healthy Taiwanese men highlighted that adherence to a pre-defined Western dietary pattern led to a decline in sperm concentration and total sperm motility^18^. Similarly, Danielewicz et al.^29^ indicated that following a Western dietary pattern, including sweets and snacks, processed meat, animal fat, refined grain products, red meat, potatoes, and dairy products, was associated with an increased risk of reduced motility, total sperm count, and abnormal sperm morphology.

On the other hand, Gaskins et al.^17^ reported no significant association between the sperm quality parameters and adherence to a Western dietary pattern, characterized by a high intake of red and processed meat, refined grains, pizza, snacks, high-energy drinks, and sweets. Although there are some null findings in the literature, the majority of previous studies have demonstrated a significant association between the Western dietary pattern and male reproductive health parameters, such as total sperm count, sperm concentration, dihydro- testosterone (DHT) 3α-diol, inhibin B/follicle-stimulating hormone ratio, and free testosterone/luteinizing hormone (LH) ratio^21,40^. Moreover, studies in this area have not particularly focused on exploring the possible association between adherence to a Western dietary pattern and fertility parameters.

The food and food groups frequently found in a diet are of great importance, as well. Eslamian et al.^23^ reported that a high intake of processed meat was positively correlated with asthenozoospermia, while many studies have suggested that a high intake of processed red meat and trans-fatty acids reduces the total sperm count and semen quality, respectively^41–43^. One possible explanation is that processed meat is rich in saturated fat, which can interact with the sperm membrane, leading to reduced sperm motility^23^. Another possible contributor is the presence of hormonal residues in processed meat^44^, because anabolic sex steroid hormones are typically injected into animals for growth promotion before slaughter^44^. It has also been reported that processed red meat contains higher concentrations of hormone residues compared to other meats, which might have an impact on the fertility status^45^.

The Western diet consists of considerable amounts of trans-fatty acids, majorly found in fast food. Previous studies have shown that a high trans-fatty acid intake reduces the serum testosterone levels, decreases the sperm count and motility, and negatively affects the normal sperm morphology^42,46^. It has also been reported that the amount of trans-fatty acids in the sperm membrane is correlated with the sperm concentration^42,46,47^, while the biological effect of trans-fatty acids yields a reduction in the activity of desaturases and a reduction in sperm count^48^.

In the present study, the highest risk of male infertility was associated with adherence to the mixed dietary pattern. Conversely, previous studies have reported a protective or an insignificant association between the mixed diet and sperm quality parameters^49,50^. The term “mixed dietary pattern” is usually used when the loaded dietary pattern consists of foods with both protective and adverse health effects if routinely consumed. Nonetheless, the nature of foods (being healthy or unhealthy relative to fertility outcomes) used in the mixed pattern determines whether it can lead to a reduced risk of infertility. This difference might be the cause of inconsistency between studies reporting a mixed dietary pattern. Based on our findings, the direct association between the mixed dietary pattern and infertility might be attributed to the animal fat and fast food, which are abundant in the mixed diet.

Likewise, regarding the inverse association between a high consumption of foods containing trans-fatty acids and the quality and quantity of sperms^42,51^, the animal fat intake seems to be negatively associated with fertility outcomes. There is accumulating evidence suggesting that a high saturated fat intake is negatively associated with sperm concentration^48^, semen volume^52^, and total sperm count^47^. It has been also shown that there is an inverse association between the monounsaturated fat-to-saturated fat ratio and prostate cancer, which might be due to the inflammatory effect of saturated fats, leading to the disruption of the endocrine system^47,53^. Besides, some lipophilic chemical residues, such as polychlorinated biphenyls and polyfluorinated chemicals, which can be found in animal products, such as organ meat, may disturb the endocrine function^54–57^. The negative association between these chemicals and semen quality has been reported in the literature^58,59^.

In the present study, the traditional dietary pattern, which consisted of large amounts of carbohydrate drinks, sweets, sugar, and deserts, was associated with a 36% increase in the risk of infertility; however, this association was not significant. Overall, foods used in the traditional diet have shown inconsistent effects on fertility, while some foods containing high amounts of sugar have shown negative effects on the sperm parameters; on the other hand, foods, such as whole grains^8^, vegetables^8^, and potatoes^60^, can have protective effects on fertility outcomes. In this regard, several studies have reported that foods, such as sweet desserts and carbohydrate drinks with a high sugar content, can reduce the volume and motility of sperms^42,43^.

However, since the Iranian traditional dietary pattern is considerably different from the habitual food choices of people in other countries, there are limited available studies for comparison. For instance, compared to Americans and Europeans, Iranians tend to consume higher amounts of potatoes, black tea, and sweet foods, such as “Gaz” and “Halva”—the foods that were heavily loaded in our traditional dietary pattern. A possible mechanism for the positive association found in the present study is that a high intake of sugar and fructose is associated with an increased insulin resistance and oxidative stress that affects sperm motility^61,62^. It seems that other foods in this dietary pattern may also affect sperm parameters, yielding a negative association, albeit insignificant.

The present study has some limitations that need to be considered. First, inherent methodological limitations to case–control study designs make it impossible to infer a causal link between dietary patterns and the risk of infertility. Second, a major concern is that participants may change their dietary options, and any marked changes can alter the risk estimates. However, we tried to minimize this limitation by selecting participants among newly diagnosed patients. Third, considering the cultural norms of Iran, the consumption of alcoholic beverages was not examined in this study; alcohol consumption is an additional factor that must be considered, particularly in non-Muslim countries and individuals; although this concern is out of the operational control of this study, it suggests a new venue for future research. Finally, although this study was conducted on a large sample size, since the participants were selected from a particular population, it is unclear whether the findings can be generalized to other communities; therefore, further investigations must consider other ethnicities to offer effective recommendations.

## Conclusion

The present study showed that adherence to a healthy dietary pattern may reduce the risk of infertility. In contrast, adherence to mixed and Western dietary patterns might be associated with the increased risk of male infertility. This study, with its large sample size, provided detailed insights into the association between diet and the risk of male infertility and demonstrated that carefully curated diets by avoiding unhealthy dietary components, could have a positive impact on reducing the risk of infertility. However, further studies are needed to confirm the accuracy of our findings.

## Materials and methods

### Study population

This age-matched case–control study was conducted on 400 males with infertility and 537 healthy individuals, referred to Yazd Reproductive Sciences Institute from July to December 2019. The participants (aged 20–55 years) were selected among newly diagnosed infertile men by an andrologist if they met at least one of the following criteria: a sperm count < 15 millions per milliliter; normal morphology < 4%; semen volume < 1.5 mL; and normal motility < 40%^63^. On the other hand, the exclusion criteria were as follows: (1) having a chronic disease, testicular atrophy, ejaculatory disorder, hypospadias, stenosis, or varicocele; (2) adherence to a specific diet which could change the subject’s habitual dietary pattern; (3) response to less than 35 items of the Food Frequency Questionnaire (FFQ); and (4) under- or over-reporting of energy intake (< 800 kcal or > 4200 kcal)^39,64^.

The control group consisted of healthy individuals without a history of infertility, which was confirmed by a seminogram in the past six months. The exclusion criteria for the control candidates were the occurrence of chronic reproductive tract diseases or hormonal abnormalities, having a family history of male reproductive disorders, following a specific diet, incomplete FFQ (response rate < 35 items), and reporting calorie intake < 800 kcal or > 4200 kcal. All subjects provided informed consent prior to participation in the study. The study protocol was approved by the Ethics Committee of Isfahan University of Medical Sciences (code: IR.MUI.RESEARCH.REC.1398.264) and conducted in accordance with the Declaration of Helsinki.

### Dietary assessment

Dietary intake was assessed using a semi-quantitative FFQ, the validity of which has been approved in Iranian populations^65^. The FFQ, including 168 food items, was designed according to the consumption frequency of the most common foods (country-specific) over the past 12 months (number of times consumed daily, weekly, monthly, and annually). The dietary assessment was conducted by a trained dietitian through a face-to-face interview. Household measurements were used to calculate the total energy and nutrient consumption (grams per day). Due to the large number of food items, 168 foods were classified into 23-pre-defined food groups for the analysis of dietary patterns; each food was assigned to a specific group, based on its nutrient similarities (Supplemental Table 2). The dietary habits of each participant were assessed one year before a confirmed infertility diagnosis. Information on alcohol use was not collected for cultural reasons and therefore not analyzed.

### Physical examination and lifestyle

The physical activity data were collected using the short form of the International Physical Activity Questionnaire (IPAQ)^66^, which was previously validated in an Iranian population^67^. The IPAQ provides data about the level, frequency (days per week), and duration of physical activity. Based on the IPAQ, the participants were categorized into inactive, moderate activity, and physically active groups if they had < 1 h/week, 1–3 h/week, and > 3 h/week of physical activity, respectively. Besides, the socioeconomic status (SES) of the participants was assessed according to the following variables: residential status (landlord/tenant), ownership of a washing machine or dishwasher (yes/no), number of annual international travels, car ownership (yes/no), individual’s occupation, and education (years of study).

### Anthropometric data, use of pesticides, and smoking data

Anthropometric data were measured according to standard methods. The hip circumference (HC) was measured at the widest circumference around the hips/buttocks, and the waist circumference (WC) was measured at the midpoint between the last rib and the iliac crest (umbilical level). The body mass index (BMI) and waist-to-hip ratio (WHR) were also calculated according to the standard protocols of the World Health Organization (WHO), based on the suggested formulae (weight [kg]/height [m^2^] and WC [cm]/HC [cm], respectively)^68^. All measurements were done while the participant was barefoot with minimal clothing. Weight was recorded to the nearest 0.1 kg, while height and circumferences were recorded to the nearest 0.1 cm, using calibrated weighing scales (Seca, Hamburg, Germany) and a stadiometer (Seca, Hamburg, Germany), respectively.

The participants were considered as pesticide users if they stored, mixed, loaded, handled, or applied any sort of agricultural or home pesticide, without wearing any personal protective equipment in the last year. Also, smoking status was assessed by asking about the habitual use of tobacco; in other words, the subjects were regarded as smokers if they had smoked more than five cigarettes per week over the past year. Other sources of tobacco were converted into the “number of cigarettes smoked per day based on the tobacco content of each source” (cigarette: 9 g of tobacco; cigar: 7.3 g of tobacco)^69^.

### Semen analysis

Before sample collection, the patients were instructed to observe 2–7 days of sexual abstinence. All semen samples were taken at the institute to avoid cooling or heating when they were transferred to the laboratory. They were kept in sterile containers at 37 °C for 30 min to complete liquefaction. Next, they were analyzed according to the WHO fifth edition of laboratory guidelines^70^. Four parameters related to semen quality, including semen volume, sperm count, normal morphology, and progressive motility, were measured.

The semen volume was assessed by either weighing or using a wide-bore volumetric pipette^71^. Regarding the sperm count, an optical phase contrast microscope was used at × 200 or × 400 magnification. The sperm morphology was also evaluated according to Tygerberg Strict Criteria^72^; the head, neck/mid piece, tail, and cytoplasmic residues of the spermatozoon were assessed to calculate the teratozoospermia index. After staining the specimens by the Papanicolaou method, adapted for human sperm morphology evaluation, assessment was performed at high magnification using a bright-field optical microscope with a 100 × high-resolution oil immersion objective lens.

Moreover, the sperm motility was assessed using a phase-contrast optical microscope (200 × to 400 ×) at 37 ± 0.5 °C by categorizing the spermatozoa as either motile (WHO classes A, B, and C, representing rapidly progressive, slowly progressive, and non-progressive spermatozoa, respectively) or immotile (class D). In this study, 10 μL of semen sample and a 2 mm × 22 mm coverslip were used to reach a depth of 20 μm. To make sure that each duplicate motility count (a minimum of 200 spermatozoa) reached a reliable value, at least five microscopic fields of view were used. All semen analyses were replicated to confirm the laboratory findings.

### Statistical methods

All analyses were performed using SPSS version 22 (IBM, Chicago, IL, USA). The normal distribution of patients’ general characteristics was determined using Shapiro–Wilk test. Quantitative variables are presented as mean ± SD, and qualitative variables as frequency (percentage). Independent samples t-test was applied to compare quantitative variables between the case and control groups, while Chi-square test was used to assess differences between categorized qualitative variables between the two groups.

To identify major dietary patterns based on 23 food groups, a factor analysis using a principal component approach (PCA) was employed. Factors were rotated using a Varimax rotation (orthogonal). The determination of retaining factors was based on eigenvalues > 1 and the Scree test. The eigenvalues markedly decreased after four factors, while the remaining factors were almost similar. Four dietary patterns were extracted and labeled according to our interpretation of data and the literature. The factor score for each pattern was determined by summing the intake of food groups, weighted by their factor loadings. Also, Kaiser–Meyer–Olkin (KMO) index, a measure of sampling adequacy (> 0.60), and Bartlett's sphericity, a test of factorability of the correlation matrix^73^, were used, which revealed that the sample size was fairly good (KMO = 0.70) and that the data were appropriate to implement a factor analysis (Bartlett’s test, P < 0.001).

Moreover, a multivariate logistic regression analysis was used to determine the relationship between dietary patterns and infertility. The analysis was performed using four different models: crude model, with no adjustment; model I: controlled for age, BMI, and physical activity; model II: adjusted for model I plus education, use of pesticides, smoking, and SES; and model III: further adjustment for energy intake. The odds ratios (ORs) and 95% confidence intervals (95% CI) were also calculated for the crude and adjusted models. A P-value < 0.05 was considered statistically significant.

### Ethical approval and consent to participate

The study protocol was approved by the Ethics Committee of Isfahan University of Medical Sciences (code: IR.MUI.RESEARCH.REC.1398.264).

## Supplementary Information

Supplementary Table 1.

Supplementary Table 2.

## Data Availability

The datasets used and/or analyzed in the current study are available from the corresponding author upon reasonable request.

## Abbreviations

BMI: Body mass index
CI: Confidence interval
FFQ: Food Frequency Questionnaire
OR: Odds ratio
PUFA: Polyunsaturated fatty acid
HC: Hip circumference
WC: Waist circumference
WHR: Waist-to-hip ratio
WHO: World Health Organization
