# Supplementary material for: The relationship between major dietary patterns and fertility status in iranian men: a case–control study
Source: Sci Rep. 2021 Sep 22;11:18861. doi: 10.1038/s41598-021-98355-4 (PMC8458458; doi:10.1038/s41598-021-98355-4)
Supplement: Supplementary file 1 — Supplementary Table 1. [file 41598_2021_98355_MOESM1_ESM.docx]

**Supplemental Table 1.** The difference of food group intake between cases and controls.

| **Food groups** | **Cases** | **Controls** | **P-value** |
| --- | --- | --- | --- |
| Red meats | 45.92±31.61 | 38.34±36.54 | <0.001 |
| Processed meats | 14.40±24.70 | 5.78±8.24 | <0.001 |
| Organ meats | 9.02±21.09 | 5.59±7.04 | <0.001 |
| Fish and other seafood | 8.71±10.42 | 30.73±38.07 | <0.001 |
| Poultry | 16.89±16.23 | 19.14±28.43 | 0.15 |
| Fast foods | 29.38±60.88 | 8.45±13.92 | <0.001 |
| Eggs | 42.89±29.13 | 46.83±52.29 | 0.13 |
| Carbonated drinks | 122.87±189 | 94.86±134 | 0.009 |
| Dairy products | 389.39±246 | 578.45±546 | 0.001 |
| Fruits and dried fruits | 459±530 | 551±610 | 0.01 |
| Vegetables | 325±190 | 367±298 | 0.01 |
| Potatoes | 26.24±17.59 | 48.47±42.21 | <0.001 |
| Legumes | 80.82±88.09 | 44.66±33.62 | <0.001 |
| Nuts | 28.04±39.93 | 41.14±67.83 | <0.001 |
| Whole grains | 143.73±136 | 121.42±207 | 0.058 |
| Refined grains | 491.25±389 | 369.08±176 | <0.001 |
| Salty snacks and vegetables | 19.38±32.99 | 29.13±64.84 | 0.006 |
| Animal fats | 6.96±10.08 | 6.05±12.61 | 0.22 |
| Vegetable oils | 23.88±17.05 | 29.79±18.12 | <0.001 |
| Olives | 3.13±6.96 | 2.19±5.24 | 0.01 |
| Sugars, sweets and desserts | 101.10±99.01 | 61.59±43.47 | <0.001 |
| Condiments and pickle | 22.36±26.68 | 43.71±51.26 | <0.001 |
| Tea and coffee | 941.42±1018 | 664.21±622 | 0.002 |

P-value was obtained from independent t-test
