# Supplementary material for: The relationship between major dietary patterns and fertility status in iranian men: a case–control study
Source: Sci Rep. 2021 Sep 22;11:18861. doi: 10.1038/s41598-021-98355-4 (PMC8458458; doi:10.1038/s41598-021-98355-4)
Supplement: Supplementary file 2 — Supplementary Table 2. [file 41598_2021_98355_MOESM2_ESM.docx]

**Supplemental Table 2.** Food groups evaluated in the dietary pattern analysis

| **Food groups** | **Food items** |
| --- | --- |
| Red meat | Beef, lamb, veal, and chopped meat |
| Processed meat | Sausage and hamburger |
| Organ meat | Liver, kidney, heart, and brain |
| Fish and other seafood | Canned tuna fish, fish, and shrimp |
| Poultry | Chicken |
| Fast food | Fried potato, French fries, and pizza |
| Eggs | Eggs |
| Carbonated drinks | Coca Cola, other carbonated beverages, and low-energy carbonated beverages |
| Dairy products | Low- and high-fat milk, yogurt and cheese, yogurt drink, chocolate milk, concentrated or creamy yogurt, ice cream, cream cheese, other cheese, and ‘Kashk’ |
| Fruits and dried fruits | Orange, tangerine, lemon, lime, grapefruit, banana, apple, pear, strawberry (and other berries), peach, cherries, fig, melon, watermelon, Persian melon, cantaloupe, raisins, grapes, kiwi, apricot, nectarine, mulberry, plum, persimmon, pomegranate, dates, tinned fruits, natural fruit juices, and other dried fruits |
| Vegetables | Cabbage, cauliflower, Brussels sprout, kale, carrot, tomato, tomato sauce, tomato paste, spinach, lettuce, cucumber, eggplant, celery, green peas, green beans, green pepper, turnip, maize, squash, zucchini, mushrooms, onions, garlic, and green leafy vegetables |
| Potatoes | Boiled potatoes |
| Legumes | Beans, peas, lima beans, broad beans, lentils, and soya |
| Nuts | Peanuts, almonds, walnuts, pistachios, hazelnuts, sunflower seeds, and roasted seeds |
| Whole grains | Iranian dark bread, barley bread, barley, and bulgur |
| Refined grains | White bread (e.g., Lavash bread and baguettes), noodles, pasta, rice, toasted bread, sweet bread, white flour, and biscuits |
| Salty snacks and vegetables | Potato chips, corn puffs, crackers, biscuits, popcorn, and pickled vegetables |
| Animal fat | Animal fat, butter, and cream |
| Vegetable oils | Vegetable oils, hydrogenated vegetable oils, mayonnaise, and margarine |
| Olives | Olives and olive oil |
| Sugar, sweet, and desserts | Sugar, candy, Iranian confectioneries (e.g., “Gaz’, ‘Sohan’, and ‘Noghl’), jam, jelly, honey, chocolate, cookies, cakes,  confections, and caramel |
| Condiments and pickles | Pepper and prickles |
| Tea and coffee | Tea and coffee |
